# Examining energy and nutrient production across the different agroecological zones in rural Ethiopia using statistical methods

**DOI:** 10.1002/fsn3.3676

**Published:** 2023-09-15

**Authors:** Habtamu Guja, Mariana Belgiu, Lidya Embibel, Kaleab Baye, Alfred Stein

**Affiliations:** ^1^ Faculty of Geo‐information Science and Earth Observation (ITC) University of Twente Enschede The Netherlands; ^2^ Center for Food Science and Nutrition, College of Natural and Computational Sciences Addis Ababa University Addis Ababa Ethiopia

**Keywords:** agroecology, antinutrients, energy and nutrient adequacy, Ethiopia, local food composition data, rural

## Abstract

Poor‐quality diets are of huge concern in areas where consumption is dominated by locally sourced foods that provide inadequate nutrients. In agroecologically diverse countries like Ethiopia, food production is also likely to vary spatially. Yet, little is known about how nutrient production varies by agroecology. Our study looked at the adequacy of essential nutrients from local production in the midland, highland, and upper highland agroecological zones (AEZs). Data were collected at the village level from the kebele agriculture office and at the farm and household levels through surveys in rural districts of the South Wollo zone, Ethiopia. Household data were acquired from 478 households, and crop samples were collected from 120 plots during the 2020 production year. Annual crop and livestock production across the three AEZs was converted into energy and nutrient supply using locally developed crops' energy and nutrient composition data. The total produced energy (kcal) met significant proportions of per capita energy demand in the highland and upper highland, while the supply had a 50% energy deficit in the midland. Shortfalls in per capita vitamin A supply decreased across the agroecological gradient from midland (46%) to upper highland (31%). The estimated shortfall in folate supply was significantly higher in the upper highlands (63%) and negligible in the highlands (2%). The risk of deficient iron and zinc supply was relatively low across all AEZs (<10%), but the deficiency risk of calcium was unacceptably high. Agroecology determines the choice of crop produced and, in this way, affects the available supply of energy and nutrients. Therefore, agroecological variations should be a key consideration when designing food system interventions dedicated to improving diets.

## Graphical abstract

Agroecology determines the choice of food produced and, in this way, affects the available supply of energy and nutrients. The nutrient composition of the same crops when grown in different agroecologies shows differences in composition. Agroecological variations should be a key consideration when designing food system interventions dedicated to improving diets.

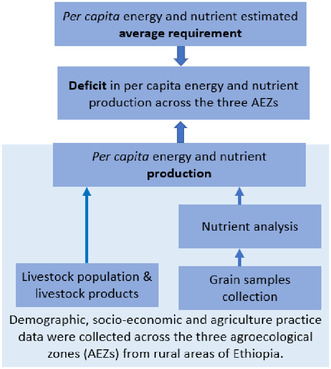

## 1 INTRODUCTION

Diets that supply the required nutrients are critical to health and well‐being. The long‐term economic and health burdens associated with poor‐quality diets are a worldwide concern. In particular, the impact on low‐ and middle‐income countries is devastating (FAO & Intake, 2022). Deficiencies of essential nutrients, both macro‐ and micronutrients, including energy, protein, vitamins, and minerals, are prevalent, particularly among children and women of childbearing age in sub‐Saharan Africa (Bailey et al., 2015; Joy et al., 2014; Mekonnen et al., 2021; Schmidhuber et al., 2018; Sheehy et al., 2019).

Such deficiencies impair normal biological function, compromise physical growth and cognitive development, increase the risk of chronic diseases, increase susceptibility to infection, and reduce productivity (Crookston et al., 2013; Gombart et al., 2020). Unlike food deprivation or overt forms of hunger, micronutrient deficiencies are commonly left unknown and thus sometimes called ‘hidden hunger’, posing a huge hurdle to the realization of various global initiatives, including the United Nations’ second Sustainable Development Goal (SDG_2) dedicated to achieving food security and improved nutrition by 2030 (Gödecke et al., 2018).

Various forms of malnutrition are the result of poor diets, inadequate knowledge and resources, and unhealthy environments, all of which have underlying causes (HLPE, 2017). Diet‐related causes are again multiple and complex, including restricted access to food, poor dietary intake, and impaired bioavailability, as well as nutrient losses due to factors such as infection (Castro‐Alba et al., 2019; Caulfield et al., 2006; HLPE, 2017).

In areas where diets are dominated by cereals and where access to foods from plant and animal sources richer in nutrients is limited, inadequate intake of energy, protein, and micronutrients, in particular iron (Fe), zinc (Zn), calcium (Ca), and vitamin A, is common (Galani et al., 2022; Gebremedhin et al., 2020; Harika et al., 2017; Kumssa et al., 2015). Most cereal grains have inherently small micronutrient concentrations (White & Broadley, 2009). However, concentrations of mineral micronutrients vary by crop type, among varieties of the same crop, and across the geographic space within countries (Bevis & Hestrin, 2021; Gashu et al., 2021; Joy et al., 2015). This variation could be attributed to the genetic make‐up, environment, agriculture management practices, and concentrations and availability of the minerals in the soil (Bevis & Barrett, 2020; Gashu et al., 2020; Ligowe et al., 2020; Manzeke et al., 2019; Reguera et al., 2018). Furthermore, cereal grains also contain large concentrations of anti‐nutritional compounds such as phytates and tannins, which inhibit the absorption of essential mineral micronutrients in the human gut (Abebe et al., 2007; Baye et al., 2014; Gibbs et al., 2011; Popova & Mihaylova, 2019).

Nutrient deficiencies occur when the foods that are available and accessible for consumption do not adequately provide the required essential nutrients. Owing to this, few studies have given attention to estimating the adequacy of available nutrients from national food production (Baye et al., 2019; Gebremedhin et al., 2020; Sheehy et al., 2019). However, such country‐level overviews have limitations in translating to fit into various contexts within the country. Heterogeneities in nutrient availability have been reported in various contexts, including rural–urban (Akerele, 2015; Mekonnen et al., 2021), across regions within a country (Baye et al., 2019; Mengistu et al., 2017; Sheehy & Sharma, 2013), and sub‐national spatial variation in crop nutrient concentrations (Abdu et al., 2022; Bevis & Hestrin, 2021; Gashu et al., 2021; Joy et al., 2015). Such micro‐level variations are of greater concern for nutrient intakes, given the widespread consumption of locally produced staple foods such as cereal and legumes among smallholder farming communities.

Agroecological zones (AEZs) limit the distribution of food production by crop type, resulting in variation in the production and availability of essential nutrients (Mihretie et al., 2022; Reguera et al., 2018; Simane et al., 2013). In this respect, agroecologically diverse countries like Ethiopia (Gebru et al., 2018) have given little research attention to evaluating nutrient production across the different AEZs, particularly in predominantly rural areas where markets are the least functional.

The aim of the present study is to identify inadequacies in nutrient availability for agroecological‐based interventions. This is achieved by (i) determining macro‐ and micronutrient concentrations of major crops growing across the three AEZs, (ii) determining the concentrations of anti‐nutrients and estimating the relative bioavailability through anti‐nutrient to mineral molar ratios, (iii) estimating the energy and micronutrient availability from local food production, and (iv) determining the proportion at risk of inadequate nutrient intake in rural villages representing different agroecologies in South Wollo, Northeastern Ethiopia.

## 2 MATERIALS AND METHODS

### 2.1 Study area

**FIGURE 1:**
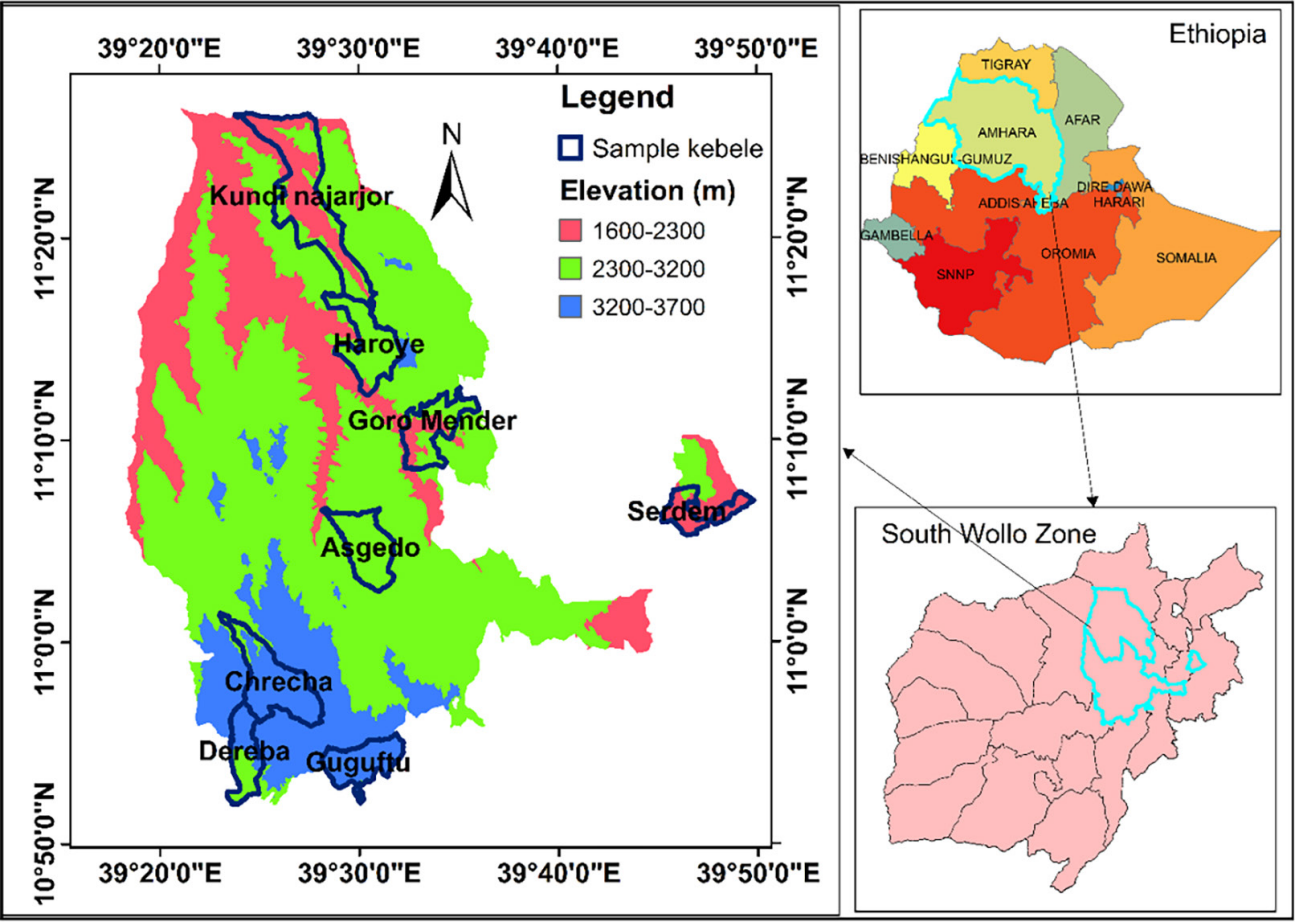
Study area disaggregated by agroecological class. Elevations in meters above mean sea level represent midland (1500–2300), highland (2300–3200), and upper highland (3200–3700).

In Ethiopia, the magnitude of malnutrition is worst in rural areas, where more than three‐fourths of the population live. A large heterogeneity occurs among regions; for instance, the Amhara region has a 46% prevalence of chronic malnutrition in children, which is higher than the national average (CSA & ICF, 2016). Likewise, Zn deficiency in the region is the highest (71.5%) among regions in Ethiopia (Belay et al., 2021). Since the region contributes to over one‐third of the national food supply (CSA, 2020), food production is likely to vary across space. The Amhara region is agroecologically diverse, with multiple agroecological zones existing within a distance of 100 km, as illustrated in the selected districts of the South Wollo zone (Figure 1). Studying this area provides an opportunity to evaluate how agroecology affects nutrient production.

### 2.2 Sampling method

Multistage sampling was used to select both study participants and plots (Chauvet, 2015). First, the two districts of the South Wollo zone were selected purposely by taking into account the representation of the major AEZs, predominantly rural villages, with accessibility considerations to allow sample collection. Second, *kebeles* (villages) falling under different AEZs were selected by means of simple random sampling. Finally, clusters within those selected *kebeles* were chosen randomly. The geographic size of the kebele, distribution of crop types, and population density were considered in determining the size of the cluster.

### 2.3 Data collection method

Data were collected at various levels in different ways: at the village level from the kebele agriculture office, at the farm level from farm owners or their representatives, and at the household level through surveys.

**FIGURE 2:**
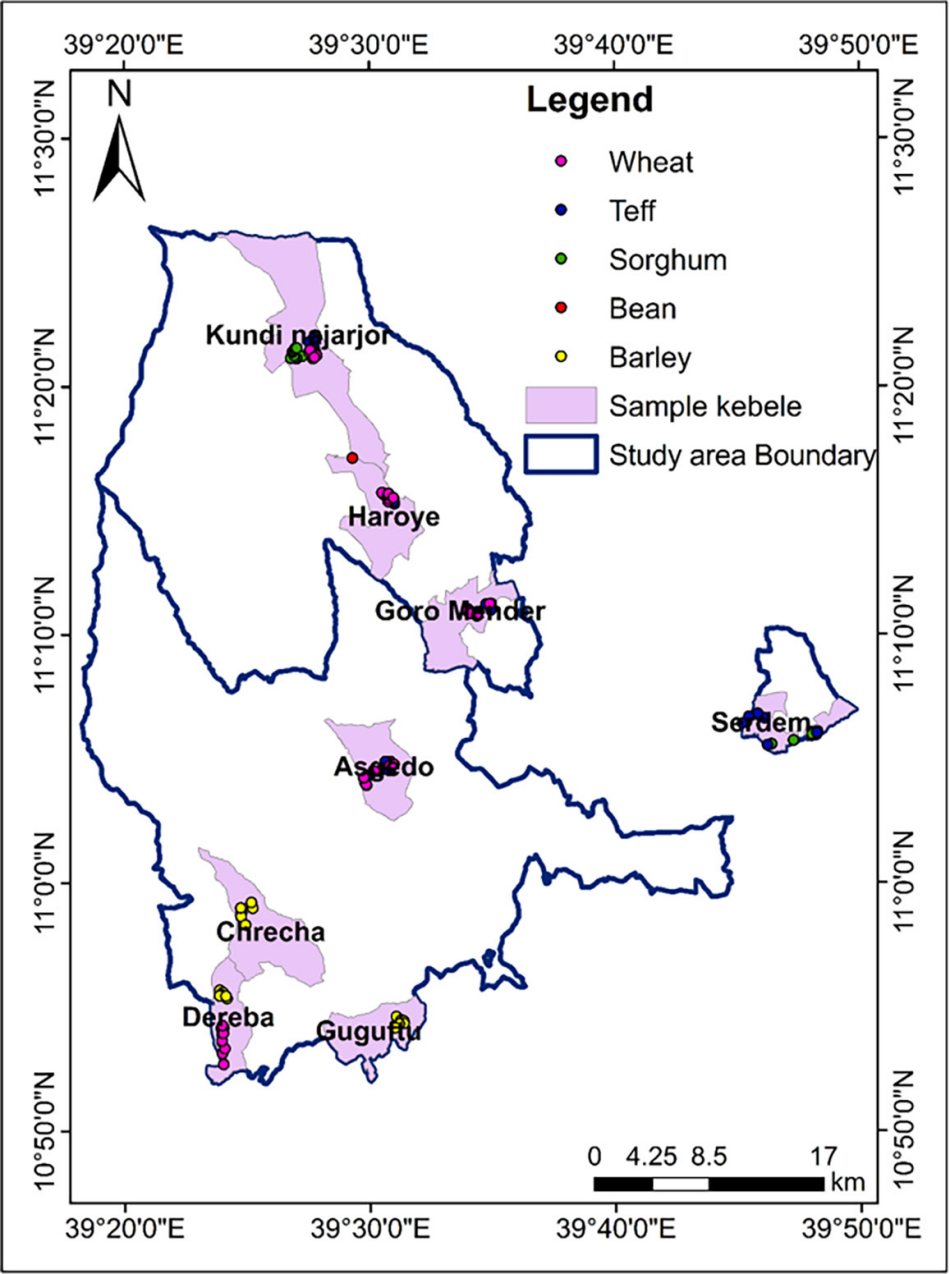
Study area map showing points where crop samples were collected.

- From a kebele agriculture office, village‐level data like the total number of registered households in the village, the total area of cultivated land, the share of cultivated land size dedicated to each crop type, the total livestock owned by type in the village, the total livestock products, and the production of minor crops including fruits and vegetables were collected. Data on agriculture management practices, the proportion of irrigation farms, cluster farming practices, type of fertilizer used and recommended application rate, average agriculture land size owned per household, etc. were also collected
- A farm‐level checklist was used to assess the agriculture management practice. Items include the practices of intercropping, crop rotation, use of organic and/or inorganic fertilizer, mode of acquiring seeds, date of sowing, expected date of harvest, size of farmland, etc. Crop samples were collected for estimating yield and determining the composition of nutrients (Figure 2). Crop yield estimation was done using the crop cut method from the subplot harvest. According to Sapkota et al. (2016), the number of subplots and area of each subplot to be selected for yield estimation through crop cuts depends on the resources available and the level of precision required in the estimation. However, in practice, 1–5 subplots with a minimum size of 1 m^2^ sampling plot have been suggested. The present study sampled three subplots per study plot, and each subplot had a harvest area of 1 m^2^.
- From the household survey, the following items were collected: data on average agriculture farmland owned by households, agriculture production season, adopted agriculture management practices, average composition of household size by age, production of homestead gardens, average livestock population owned by type, proportions of households producing livestock products, and average livestock production per household (eggs, milk, and honey).

Sampling was done in November and December of 2020, i.e., from the mid‐ and highland kebeles during the *Meher* production, and in mid‐June until the end of July 2020, during the *Belg* barley harvesting in the upper highland. The household survey was conducted from March 7 to April 19, 2021.

#### 2.3.1 Field crop sampling considerations

During the planning stage of grain sample collection for major crops, the following points were considered regarding sampling strategy and density: to collect enough samples in a small area, Congalton and Green (2019) suggest the importance of setting a minimum number of samples in each class and then adding additional samples to each class proportionally to the geographic scale of the crop's distribution. When the geographic distribution of crop types differs, sampling becomes more intensive in some regions than in others (Foody et al., 2016). In addition, when the inherent variability of the plots within a cluster is minimal, minimum numbers of samples are collected.

The study used zone‐based sampling applied to field sub‐division (Muhammed et al., 2017). This sampling method is more cost‐effective than grid‐based sampling in smallholder farming with diverse crop cultivation when complemented with stratification and randomization (Mallarino & Wittry, 2004). It is assumed to capture variations in nutrient concentration over smaller spatial scales arising from variations in agriculture management practices (Lawrence et al., 2020; Mallarino & Wittry, 2004). Hence, farm plots were stratified at each cluster by crop type(s) across comparable elevations, from which study plots were randomly selected. Crop samples were then collected from farm plots.

From the selected plots, three sub‐samples were taken across the diagonal line – as practically as possible from the different crop rows – with a caution to avoid adjacent plot effects (edge effect), as depicted in Figure S1. Samples from the selected plots were taken from three subsamples, each yielding a 1 m^2^ quadrant harvest for all the crops except sorghum. For sorghum, average heads of the grain found within the three 1 m^2^ quadrants were identified first, followed by averaging the number of heads that were randomly and proportionally taken from each of the three sub‐sample quadrants. The harvested sub‐samples per plot were aggregated to form a composite sample and kept in a sample bag until sample preparation.

Grain samples were thus collected from 120 major crop samples for different crop types, including teff (*n* = 30), wheat (*n* = 30), sorghum (*n* = 24), beans (*n* = 18), and barley (*n* = 18). Unlike the other staple crops, sorghum has been growing only in two warmer kebeles, thus allowing for twice as many samples per cluster for nutrient analysis. Equal proportions of teff (*Eragrostis tef* (*Zuccagni*) *Trotter*) and wheat (*Triticum aestivum L*.) were collected from mid‐ and highland. Whereas sorghum (*Sorghum bicolor (L.) Moench*) and beans (family *Fabaceae*) were collected, respectively, from midland and highland, barley (*Hordeum vulgare L*.) samples were exclusively collected from the upper highlands.

### 2.4 Sample preparation

The composite harvests were manually threshed in the Centre for Food Science and Nutrition laboratory, Addis Ababa University. Unlike field threshing, such manual threshing minimizes extrinsic mineral contamination occurring from soil/dust, as indicated by Guja and Baye (2018). Additionally, Gashu et al. (2021) indicated grain concentrations of Ca and Fe are more sensitive to soil dust due to the much higher total concentrations of these elements in soils, as shown from studies in Ethiopia and Malawi. Whole‐grain samples were air‐dried in their sample bags. Each sample was then ground in a stainless‐steel grinder (Xian Siway Scientific Instrument, Model: ZN‐08, Japan), which was wiped clean before use and after each sample with a non‐abrasive cloth.

All the sample preparations were done away from sources of contamination by soil or dust, and the sample preparations followed the protocols for micronutrient analysis by Stangoulis and Sison (2008). Finally, 20 g of ground subsamples were prepared and made available for laboratory analysis of desired parameters at (i) Center for Food Science and Nutrition – at the Addis Ababa University, (ii) Ethiopian Food and Drug Authority (EFDA), (iii) Ethiopian Public Health Institute (EPHI), and (iv) Debrezeit Agriculture Research Centre.

### 2.5 Grain nutrient analyses

Moisture, protein, fat, and ash were analyzed according to the methods of the Association of Official Analytical Chemists (AOAC, 2000, 2005, 2007). Moisture content was determined by oven drying at 105°C to a constant weight (protocol no: AOAC. 925.10). Crude fat content was determined using the Soxhlet method (AOAC 991:36). Protein content was determined by the Kjeldahl method based on the determination of nitrogen content (AOAC. 981:10). The crude fiber contents of the samples were determined following the method indicated in AOAC (2007). Ash was determined gravimetrically in a heated muffle furnace at 550°C. Total and available carbohydrate contents were computed by difference, as indicated in Eq. (1a) and (1b):

(1a) $$\%\text{Total carbohydrate}=100-\left(\%\text{Moisture}+\%\text{Crude protein}+\%\text{Crude}\;\mathrm{fat}+\%\mathrm{Ash}\right)$$

(1b) $$\%\text{Available carbohydrate}=100-\left(\%\text{Moisture}+\%\text{Crude protein}+\%\text{Crude}\;\mathrm{fat}+\%\mathrm{Ash}+\%\text{Crude fiber}\right)$$

The gross energy value, expressed in kilocalories (kcal), was calculated using Atwater's conversion factors of 4 kcal/g for protein, 9 kcal/g for fat, 4 kcal/g for carbohydrates, and 2 kcal/g for fiber (FAO, 2003; Slavin & Carlson, 2014) (Eq. (2)).

(2) $$\text{Gross energy}\;\left(\text{kcal}\right)=\left[\left(9\times \text{Crude}\;\mathrm{fat}\right)+\left(4\times \text{Crude protein}\right)+\left(4\times \text{available carbohydrate}\right)+\left(2\times \text{Crude fiber}\right)\right]$$

Mineral micronutrient analysis was done using a Microwave Plasma‐Atomic Emission Spectrometer (MP‐AES 4200, Agilent Technologies) following wet ashing. Certified reference materials (CRM) were used to construct a calibration curve for each mineral determined. Finally, the determinations of anti‐nutrients were carried out following the methods described in Vaintraub and Lapteva (1988) for phytates and Butler et al. (1982) for tannin. The vitamin C assay method was used to determine the vitamin C concentration of crop samples using a UV‐spectrophotometer.

### 2.6 Estimates of agriculture production and nutrient supply

The crop output for *meher* (the long rainy season and the main cropping season) and *belg* (the short rainy season) was collected from agriculture field surveys and the village/kebele agriculture office to generate an annual estimate of crop production. Estimates of the livestock population and livestock products were obtained from household surveys conducted in selected villages representing the three AEZs. The total annual production of each crop in each agroecological region was aggregated. For livestock products, the total annual production of cow milk, eggs, and honey was considered.

Annual crop and livestock production was then translated into energy and seven different micro‐ and macronutrients using local food composition data from our laboratory analysis. For nutrients that were not analyzed, values were borrowed from the Ethiopian food composition tables (ENI, 1998), the US Department of Agriculture nutrient database (USDA, 2016), and Baye et al. (2021), particularly for livestock products. The choice of these nutrients was based on their biological importance in the physical growth and cognitive development of children and the well‐being of the general population. The energy and nutrients produced were expressed on a per‐day basis to enable comparison with daily requirements by dividing by 365 using the population size for that particular year.

### 2.7 Energy and nutrient requirements

To estimate dietary requirements, the estimated average requirement (EAR) for the study area was calculated for each nutrient using data from WHO/FAO (2004) and the Institute of Medicine (IOM, 2001, 2002). The EARs were derived from the reference nutrient intake (RNI), which is the intake level sufficient for approximately 97.5 percent of a specific sex and life‐stage group. RNIs provided by WHO/FAO and IOM were converted to EARs using standard conversion factors (WHO, 2006).

Sex‐ and life‐stage‐specific EARs were calculated using population estimates disaggregated by sex and age. The projected population size of the Amhara region by age group and sex for the year 2021 was used to compute the proportion of rural inhabitants in each study village by age group (CSA, 2013).

The crude pregnancy rate was calculated for each age group as crude birth rate (CBR) × 280/365, assuming pregnancy lasts for 280 days. The proportion of lactating women was calculated by assuming that breastfeeding was continued until two years (CBR × 2), as previously described in Joy et al. (2014). The EAR for Ca was set by assuming a low animal protein intake. The EARs for Fe and Zn across the life stages, including during pregnancy and lactation, were estimated assuming low bioavailability as provided in WHO/FAO (2004). Nutrient requirements by age, sex and life stages are provided in the Data S2.

### 2.8 Estimating intake distribution and prevalence of nutrient production deficits

The EAR cut‐off point method is used to estimate the prevalence of food production deficits (Beaton, 1994). We estimated a population distribution around the mean estimated intake per capita, which was derived from the food production data for each nutrient. This was done by calculating a coefficient of variation (CV) of intake based on within‐subject variation from values obtained from published literature (Beal et al., 2017). The CVs used were as follows: energy, protein, zinc, and calcium (CV = 0.25), vitamin A (CV = 0.45), and vitamin C and Fe (CV = 0.4). A nutrient intake distribution was assumed to be normally distributed for CV values 0.3 or lower and log‐normally if the CV was greater than 0.3. We applied this CV to obtain a distribution of estimated micronutrient intakes across the AEZs. The proportion of the population below the EAR was considered to estimate the production deficits.

### 2.9 Statistical analysis

Crop nutrient analyses were carried out in duplicate. If the coefficient of variation between two successive measurements for each crop sample exceeded 5%, then measurements were repeated. The results were expressed as the mean ± SD. Data normality was checked using the Shapiro–Wilks test. If the data did not violate the assumption of normality, a one‐way and two‐way analysis of variance (ANOVA) was carried out comparing variables across the three AEZs. The two‐way ANOVA was used to investigate the combined effect of crop types and agroecological classes on nutrient composition, anti‐nutrients, and phytate‐to‐mineral molar ratios. Independent sample t‐testing was used to compare means between two comparison groups. If the null hypothesis of normality was rejected, a non‐parametric Mann–Whitney *U* test or Kruskal–Wallis test was used to compare means between two and among three comparison groups, respectively. Associations between two categorical variables were examined using the Chi‐square test. Statistically significant differences were considered at a *p*‐value less than .05, while means separation was done by the Duncan post hoc test. SPSS version 28 was used to analyze the data.

### 2.10 Compliance with ethical standards

Household surveys and field crop samples were collected after obtaining informed consent from the farm owners/representatives. Additionally, the farmers were compensated for the crop sample harvest with a reasonable estimate of the market price. The work was conducted under ethical approval from the College of Natural and Computational Sciences, Addis Ababa University (Reference No: CNSDO/185/12/19, Dated 13/11/2019).

## 3 RESULTS

### 3.1 Agriculture production‐related characteristics of households

Crop and livestock‐mixed farming is practiced across all the studied AEZs. Variations exist in the levels of livestock ownership and types of crops produced across the AEZs (Table 1). Twice as many households in the highlands own milk cows as in the midlands. Likewise, differences have been observed in the chicken population between highland (61.45%) and upper highland households (29.2%). The mean productivity of milk (liters/cow/day) is significantly higher in the upper highland (2.5) than in the midland (1.6) and highland (1.5).

**TABLE 1:** Agriculture production‐related characteristics of households from the different agroecological zones in South Wollo, Ethiopia.

Variables	Agroecological class			*p*‐value
	Midland (*n* = 175)	Highland (*n* = 166)	Upper highland (*n* = 137)	
Major crops				
Major crops cultivated	Teff, wheat, sorghum	Bean, teff, wheat	Barley	
Mean livestock population owned per HH^†^				
Cattles	1.30 ± 0.10^a^	**1.83** ± 0.10^b^	1.27 ± 0.11^a^	**.001**
Milk‐cow	0.35 ± 0.04^a^	0.64 ± 0.05^b^	0.47 ± 0.05^a^	**.001**
Sheep	0.20 ± 0.05^a^	**3.43** ± 0.29^c^	1.78 ± 0.21^b^	**.001**
Goat	1.03 ± 0.14^b^	1.06 ± 0.19^b^	0.33 ± 0.07^a^	**.001**
Chicken	1.63 ± 0.15^a^	**3.27** ± 0.38^b^	1.71 ± 0.26^a^	**.001**
Beehives	0.17 ± 0.04^a^	0.42 ± 0.18^a^	0.03 ± 0.02^a^	.055
Livestock products				
Proportion of HH producing cow milk	15.43%	33.13%	24.09%	
Volume of milk (Liter/cow/day)^†^	1.62 ± 0.07^a^	1.46 ± 0.07^a^	**2.52** ± 0.17^b^	**.001**
Proportion of HHs having egg‐lying chicken	44.0%	61.45%	29.20%	
Quantity of eggs laid per HH per day^†^	2.49 ± 0.11^a^	3.43 ± 0.36^a^	2.45 ± 0.27^a^	.143
Proportion of HHs producing honey	10.29%	9.64%	1.46%	
Honey production (kg/year)^†^	4.22 ± 0.36^a^	4.25 ± 0.38^a^	2.50 ± 0.18^a^	.491
Agriculture land size owned^†^				
Land size owned in ha (Mean ± SD)	0.52 ± 0.03^a^	0.76 ± 0.03^b^	0.8 ± 0.07^b^	**.001**
Non‐farm household income (%)				
Yes	45 (27.3)	32 (20.4)	31 (23.5)	.347
No	120 (72.7)	125 (79.6)	101 (76.5)	
Mode of acquiring seed (%)				
Purchasing improved seed	21 (15.4)	30 (19.1)	19 (19.4)	.165
Local/landraces from own produce	115 (84.6)	127 (80.9)	79 (80.6)	
Use of inorganic fertilizer (%)				
Users	118 (81.4)	137 (86.2)	23 (23.0)	**.001**
Non‐users	27 (18.6)	22 (13.8)	77 (77)	
Practice of crop rotation (%)				
Yes	126 (87.5)	140 (92.1)	14 (12.3)	**.001**
No	18 (12.5)	12 (7.9)	100 (87.7)	
Practice of intercropping (%)				
Yes	40 (26.7)	40 (25.6)	7 (6.1)	**.001**
No	110 (73.3)	116 (74.4)	107 (93.9)	
Adopted agriculture production season (%)				
*Meher only*	142 (81.1)	132 (79.5)	10 (7.3)	**.001**
*Belg only*	1 (0.6)	23 (13.8)	75 (53.7)	
Both *Meher* and *Belg*	32 (18.3)	11 (6.6)	52 (38.0)	
Practice of irrigation farming (%)				
Present	14 (9.8)	10 (6.5)	9 (8.1)	.591
Absent	129 (90.2)	143 (93.5)	102 (91.9)	

*Note*: Chi‐square test was used to compare categorical variables *n* (%). Bold values indicate significant at 95% confidence interval.

Abbreviation: HH, household.

^†^ Values in mean ± standard error, computed using mean comparison, where means not followed by the same superscript letters across the row are significantly different (*p* < .05) from each other.

Rain‐fed farming is the dominant form of agriculture. Across all the AEZs, only 30% of the studied households own farmland size of ≥1 ha. While the average farmland size in the highlands and upper highlands is 50% greater than in the midlands. Across all the AEZs, the majority of farming households obtain seeds from local/own production. More than 80% of the households in midland and highland utilize inorganic fertilizers and practice crop rotation, whereas in the upper highland, only 23% of the households reported utilization of inorganic fertilizers and crop rotation is negligibly practiced owing to barley monocropping (Table 1).

### 3.2 Proximate composition

Table 2 shows that the broad bean has the largest crude protein (23.6 g/100 g) and crude fiber content (12.18 g/100 g). Sorghum provides the highest crude fat (3.22 g/100 g) and available carbohydrate (73.23 g/100 g) among cereal crops (Table 2).

**TABLE 2:** Proximate composition of major crops grown in Dessie Zuria and Kutaber districts of the South Wollo zone, Ethiopia.

Crop types	Parameters of proximate composition (g/100 g, DWB)					Gross energy (kcal/100 g)
	Crude protein	Crude fat	Crude fiber	Total ash	Available carbohydrate	
Teff	8.81 ± 1.01^b^	3.03 ± 0.77^c^	5.66 ± 3.53^a^	2.59 ± 0.43 ^b^	71.48 ± 4.42 ^bc^	361.12 ± 12.18 ^b^
Wheat	10.10 ± 1.66 ^c^	2.42 ± 0.48^bc^	5.44 ± 1.50 ^a^	1.83 ± 0.15 ^a^	71.40 ± 2.68 ^bc^	359.37 ± 6.33 ^b^
Bean	23.59 ± 1.87 ^d^	1.68 ± 0.51 ^a^	12.18 ± 3.17 ^c^	2.47 ± 0.89 ^b^	52.21 ± 4.32 ^a^	344.34 ± 8.66 ^a^
Barley	8.06 ± 0.67 ^ab^	1.84 ± 0.65 ^ab^	8.91 ± 2.82^b^	2.60 ± 0.47 ^b^	69.63 + 2.44 ^b^	345.73 ± 5.29 ^a^
Sorghum	7.45 ± 1.26^a^	3.22 ± 0.86^d^	5.90 ± 2.13 ^a^	2.16 ± 0.37^ab^	73.23 ± 2.41 ^c^	362.97 ± 4.52 ^b^
*p*‐value	<.001	<.001	<.001	<.001	<.001	<.001

*Note*: Data are expressed as the mean ± SD. Means that do not share the same letter down the column are significantly different.

Abbreviation: DWB, dry weight basis.

Regarding constituents of gross energy, beans provide 60% of energy from carbohydrates, whereas, in the case of teff, wheat, barley, and sorghum, at least 70% of gross energy is composed of carbohydrates. On the contrary, protein‐energy from beans accounts for about 27.4%, and the rest of cereal crops provide much lower amounts: sorghum (8.2%), barley (9.4%), teff (9.8%), and wheat (11.4%).

### 3.3 Mineral contents

Table 3 presents mineral micronutrient concentrations among major crops. It shows that teff is relatively the highest in both iron and zinc concentrations, followed by barley, whereas sorghum is the lowest in both iron (3.31) and zinc (1.78) concentrations in mg/100 g.

**TABLE 3:** Micronutrient concentrations of major crops grown in Dessie Zuria and Kutaber districts of the South Wollo zone, Ethiopia.

Crop types	Concentrations of minerals and vitamin C (mg/100 g)			
	Iron (Fe)	Zinc (Zn)	Calcium (Ca)	Vitamin C
Teff (*n* = 30)	11.21 ± 6.05^c^	3.44 ± 1.19^d^	30.69 ± 13.47^c^	0.529 ± 0.181^a^
Wheat (*n* = 30)	3.79 ± 2.26^a^	2.38 ± 1.00^b^	8.79 ± 6.70^a^	0.864 ± 0.144^b^
Bean (*n* = 18)	4.20 ± 0.63^a^	2.74 ± 0.25^bc^	29.81 ± 8.72^c^	1.346 ± 0.549^c^
Barley (*n* = 18)	8.36 ± 2.94^b^	3.05 ± 1.11 ^cd^	13.26 ± 1.09^a^	0.476 ± 0.141^a^
Sorghum (*n* = 24)	3.31 ± 0.64^a^	1.78 ± 0.44^a^	23.33 ± 11.08^b^	0.475 ± 0.371^a^
*p*‐value	<.001	<.001	.001	<.001

*Note*: Data are expressed as the mean ± SD. Means that do not share the same superscript letters down the column are significantly different from each other.

### 3.4 Anti‐nutritional factors

Crop types have significant variabilities in the content of antinutrients. Teff has the highest phytate concentration but the lowest tannin concentration. Bean and barley have relatively lower contents of phytate. The tannin content of sorghum is twice as high as the concentration in teff (Table 4).

**TABLE 4:** Antinutrients concentrations of major crops grown in Dessie Zuria and Kutaber districts of the South Wollo zone, Ethiopia.

Crop types	Parameters of anti‐nutritional factors (mg/100 g, DWB)	
	Phytates^†^	Tannin
Teff	363.69 ± 87.86 ^c^	89.62 ± 14.39 ^a^
Wheat	255.64 ± 36.08 ^b^	98.11 ± 19.91 ^a^
Bean	223.63 ± 15.75 ^a^	128.07 ± 13.44 ^b^
Barley	223.34 ± 35.73 ^a^	102.05 ± 6.69 ^a^
Sorghum	251.26 ± 31.53 ^b^	148.54 ± 27.76 ^c^

*Note*: Data are expressed as the mean ± SD. Means that do not share the same superscript letter down the column are significantly different.

Abbreviation: DWB, dry weight basis.

^†^ At 90% confidence interval.

#### 3.4.1 Relative mineral bioavailability and molar ratios

**FIGURE 3:**
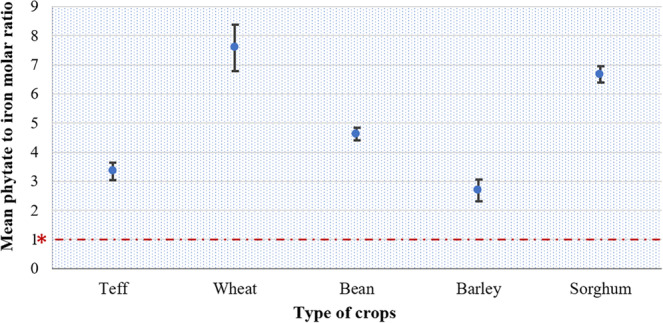
Phytate‐to‐Fe molar ratios for major crops grown in selected districts of South Wollo, Ethiopia. Error bars represent the standard error of the means. *Molar ratio values >1 indicate poor estimated bioavailability of Fe.

Figure 3 presents the mean phytate to Fe molar ratios for all the study crop types. Among the studied crops, higher mean values were reported in wheat (7.59) and sorghum (6.67), whereas teff and barley were presented with significantly lower mean ratio values, 3.34 and 2.68, respectively (*p* < .001). For all the study crops, phytate‐to‐iron molar ratio values were higher than the desired critical value of <1 (Hurrell & Egli, 2010; Magallanes‐López et al., 2017).

**FIGURE 4:**
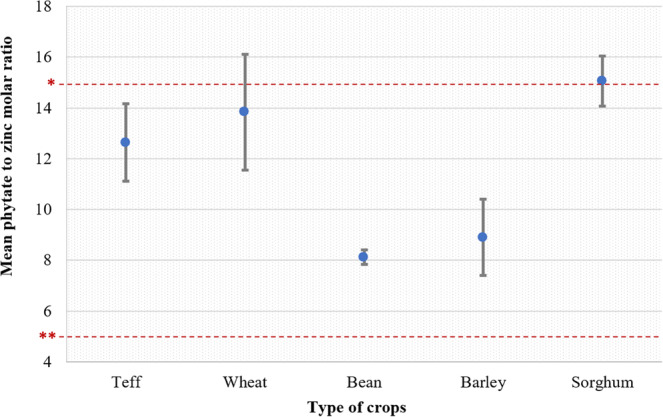
Phytate to Zn molar ratios for major crops grown in selected districts of South Wollo, Ethiopia. Error bars represent the standard error of the means. *Molar ratio value >15 indicate poor bioavailability. **Molar ratio value <5 indicate high estimated bioavailability.

The phytate‐to‐Zn molar ratio of crops presented significant variability (*p* = .018). Compared with other crops, beans and barley presented significantly lower mean ratio values, 8.13 and 8.91, respectively (*p* = .018). A phytate‐to‐Zn molar ratio > 15 is associated with low bioavailability, while ratio values between 5 and 15 are associated with moderate bioavailability (WHO/FAO, 2004). Hence, with the exception of sorghum, the mean ratio values of all crops fell under the category of moderate Zn bioavailability (Figure 4).

### 3.5 Concentrations of nutrients and antinutrients across the agroecological zones

Among the major crops grown in selected districts of South Wollo, teff and wheat grow both in the mid‐ and highland AEZs. However, other crops, including barley, bean, and sorghum are mainly grow in a particular agroecological class that is in the upper highland, highland, and midland, respectively. The nutrient compositions of two crops, teff and wheat, grown in two AEZs were compared (Table 5). In wheat, the protein content was significantly higher in the midland, whereas the ash content was higher in the highland AEZs. In the case of teff, the phytate concentration was significantly higher in the midland AEZ.

**TABLE 5:** Nutrient, antinutrient concentrations and relative bioavailability of minerals in wheat and teff grown across the different agroecological zones of South Wollo, Ethiopia.

Parameters	Wheat			Teff		
	Midland	Highland	*p*‐value	Midland	Highland	*p*‐value
Proximate (g/100 g)						
Ash	1.75 ± 0.013	1.87 ± 0.18	**.044**	2.48 ± 0.37	2.74 ± 0.46	.106
Fiber	5.04 ± 1.05	5.72 ± 1.72	.276	4.50 ± 2.47	7.11 ± 4.19	.054
Protein	11.36 ± 2.37	9.47 ± 0.68	**.032**	8.43 ± 0.91	9.49 ± 0.90	.059
Fat	2.25 ± 0.45	2.51 ± 0.47	.174	3.29 ± 0.56	2.46 ± 1.18	**.024**
Minerals (mg/100 g)						
Iron	3.39 ± 1.95	4.06 ± 2.47	.477	11.93 ± 6.56	10.31 ± 5.47	.501
Zinc	2.33 ± 1.20	2.42 ± 0.89	.817	3.60 ± 1.05	3.24 ± 1.36	.434
Calcium	10.98 ± 7.21	5.49 ± 4.35	**.042**	35.44 ± 15.76	24.76 ± 6.61	**.038**
Antinutrients (mg/100 g)						
Tannin	93.35 ± 16.74	100.97 ± 21.62	.375	86.37 ± 12.63	93.69 ± 15.93	.194
Phytate	258.43 ± 27.13	253.78 ± 41.82	.760	398.97 ± 60.93	319.59 ± 98.50	**.016**
Phy: Mineral MR						
Phy: Fe	7.51 ± 2.41	7.64 ± 4.87	.938	3.58 ± 1.71	3.04 ± 1.27	.374
Phy: Zn	16.64 ± 16.74	11.97 ± 5.76	.326	13.00 ± 8.62	12.18 ± 7.27	.795
Phy: Ca	2.42 ± 2.09	5.25 ± 4.07	**.031**	0.84 ± 0.41	0.85 ± 0.41	.915

*Note*: Data are expressed as mean ± SD. *p*‐values marked in bold indicate significant group differences.

Abbreviations: MR, molar ratio; Phy: Fe, phytate‐to‐iron molar ratio; Phy: Zn, phytate‐to‐zinc molar ratio.

Even though the phytate‐to‐Zn molar ratio of wheat did not present a statistically significant difference between the two AEZs, nutritionally relevant differences were observed. The mean molar ratio value in midland (16.64) was above the acceptable critical cut‐off (i.e., <15) than the value reported in highland (11.97).

**FIGURE 5:**
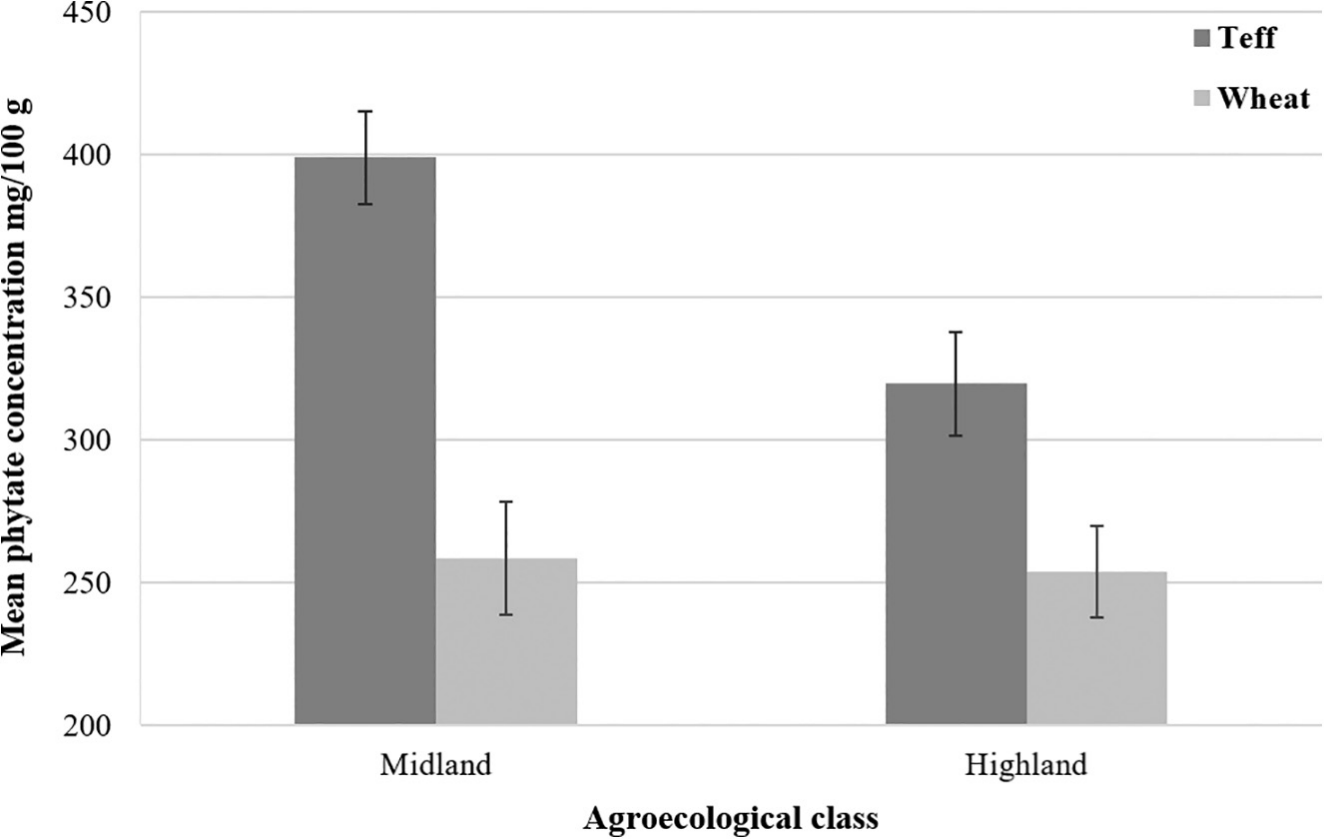
Mean phytate concentrations of wheat and teff grown across the mid‐ and highland agroecological classes. Error bars represent the standard error of the means.

In the case of teff, statistically significant interactions between crop phytate content and agroecological class were observed (Figure 5). The concentration of phytate (mg/100 g) was significantly higher in the midland (398.97 ± 16.24, 95% CI = 374.42–423.51) than in the highland agroecological zone (319.59 ± 18.15, 95% CI = 292.15–347.03). Crop type explains 41.5% of the variability in phytate concentration (*p* < .001), 10.5% in the agroecological zone (*p* = .022) and 8.5% in their interaction (*p* = .04).

### 3.6 Energy and nutrient production

Energy and nutrient production showed variabilities in magnitude across the agroecological zones (Table 6). Per capita energy production in the midland is 54.55% lower than that of the highland and by far lower than that of the upper highland. Carbohydrates contribute 77% or more to the total per capita energy production across all the AEZs. In comparison to the midland, vitamin A production in the highland and upper highland is two‐ and three‐fold higher, respectively. Per capita production of folic acid is 50% or higher in the highlands than in the other AEZs.

**TABLE 6:** Energy and nutrient per capita production across the agroecological zones in South Wollo, Ethiopia.

	Midland	Highland	Upper highland	EAR
Energy (kcal)	2234.3	3453	4431.6	2869.7
Protein (g)	62.7	98.6	107.8	51.9
Fat (g)	20.1	27.5	30.4	85.5
Carbohydrate (utilizable) (g)	431.8	667.8	873.7	148.1
Fiber (g)	37.5	68.8	112	31.5
Calcium (mg)	169	196.6	250.7	700.1
Iron (mg)	35.9	57.3	105.5	12.1
Zinc (mg)	15.4	26.1	38.7	11.3
Vitamin A (μg RAE)	36.5	86.6	146.2	625.6
Folic acid (μg)	363	541.2	296.7	329.9
Vitamin C (mg)	5.1	9.4	8.9	38.6

*Note*: Values are production.

Abbreviation: EAR, estimated average requirement.

Table 7 presents the percent deficit in per capita energy and nutrient production. The total energy (kcal) produced met significant proportions of per capita energy demand in the highland (80%) and upper highland (98.5%), whereas nearly 50% of the energy deficit is estimated in the midland. The risk of vitamin A deficiency was shown to decrease across the agroecological gradient: midland (46%), highland (39%), and upper highland (31%). The per capita deficit in folate is highest in the upper highlands and negligible in the highlands. Fe and Zn requirements are nearly met through local production, with the percent deficit remaining below 10% in all the AEZs. However, the risk of Ca deficiency is significantly high in all AEZs.

**TABLE 7:** Percent deficit in per capita energy and nutrient production relative to EAR across the different agroecologies in South Wollo, Ethiopia.

	Midland	Highland	Upper highland	EAR
Energy (kcal)	48.8	20.8	1.5	2869.7
Protein (g)	20.3	0.2	0.0	51.9
Fat (g)	99.9	99.7	99.5	85.5
Carbohydrate (utilizable) (g)	0.0	0.0	0.0	148.1
Calcium (mg)	99.4	99.2	98.4	700.1
Iron (mg)	3.2	8.0	3.6	12.1
Zinc (mg)	7.7	1.0	2.6	11.3
Vitamin A (μg RAE)	45.8	38.8	31.0	625.6
Folic acid (μg)	36.9	1.6	63.1	329.9
Vitamin C (mg)	46.2	35.5	36.8	38.6

*Note*: Values are percent deficits in production relative to EAR.

Abbreviation: EAR, estimated average requirement.

**FIGURE 6:**
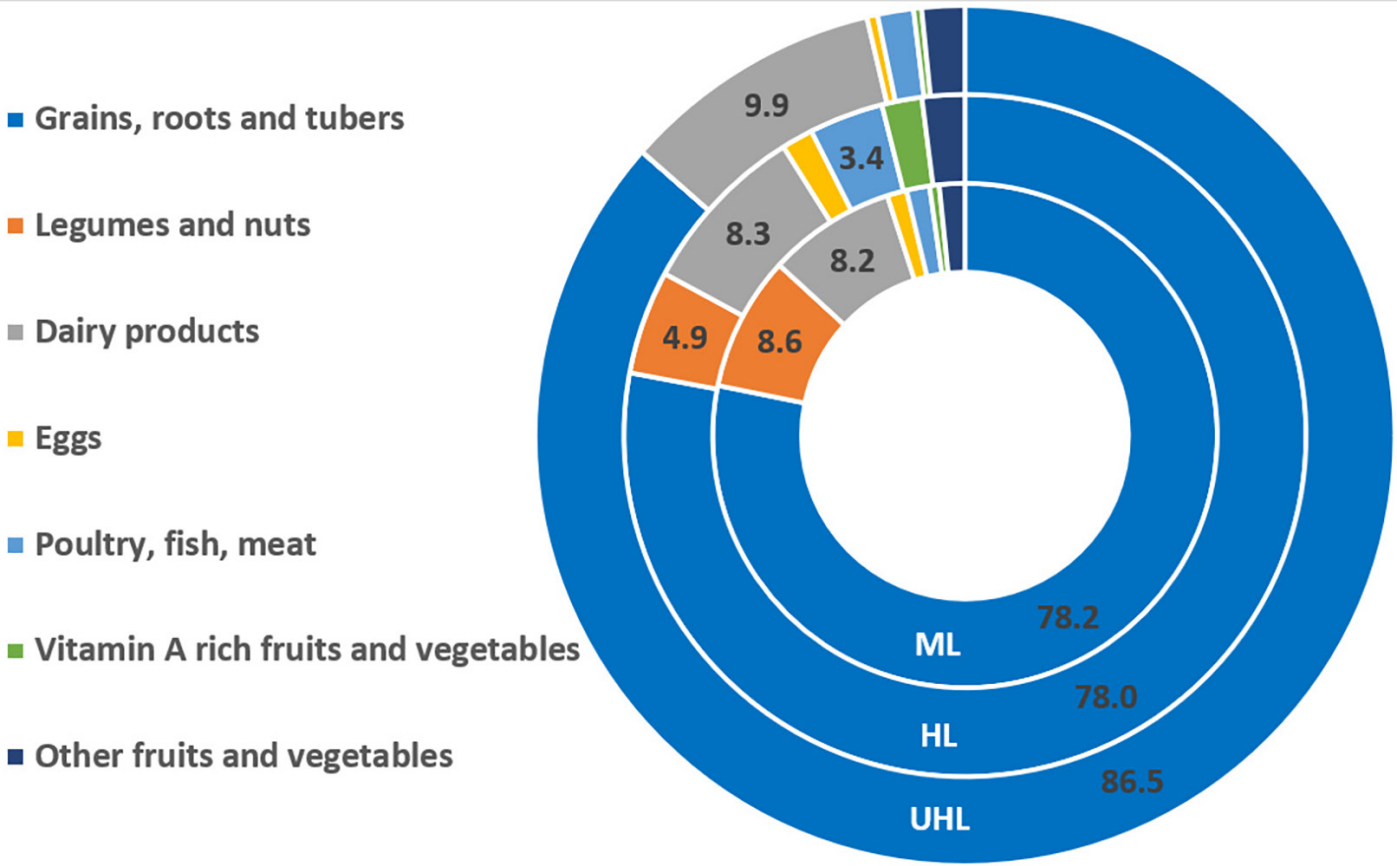
Per capita supply by food group per day from agricultural production in each agroecological zone. Note: Food groups whose values are not labeled represent those with an aggregate contribution of <5%. ML, Midland, HL, Highland, and UHL, Upper highland.

Figure 6 depicts local productions across the AEZs into nutritionally relevant seven food groups (WHO, 2008). The use of such a similar dietary diversity scale facilitates the comparison of food groups produced across different AEZs (Gupta et al., 2020). Hence, production is generally dominated by cereals. Legumes and nuts are missing in the upper highlands. The contribution from dairy products increased along the agroecological gradient. Compared with the other food groups, the aggregate contribution of flesh foods and vitamin A‐rich fruits and vegetables is less than 6% of per capita food production. For instance, for every 100 g of food produced in the midland AEZ, the relative per capita contribution by food group equates to cereals (78.2 g), legumes (8.6 g), and dairy products (8.2 g), whereas the aggregate contribution of the remaining four food groups is only 5 g.

## 4 DISCUSSION

The present study estimated energy and nutrient production across the different AEZs of Ethiopia using locally developed food composition data. The total energy (kcal) produced met significant proportions of the per capita energy demand in the highlands and upper highlands. However, the midland is estimated to have a nearly 50% energy deficit. The per capita risk of vitamin A deficiency was shown to decrease across the agroecological gradient. The risk of folate deficiency is significantly higher in the upper highlands and negligible in the highlands. The risk of Fe and Zn supply shortfalls was low across all AEZs. However, the available supply of minerals is shown to be greatly affected by antinutrients. Contrasting the other minerals, the nutrient supply side deficiency of Ca is unacceptably high.

Unlike the other AEZs, nearly half of the midlanders are at risk of a per capita energy deficit. This could be partly attributed to the major crop, teff, which is dominantly cultivated and has a lower relative yield than other crops. Teff is cultivated both in the midland and highland; however, the share of cultivated land dedicated to teff in the midland is higher (44%) than that of the highland (18%) from the total cultivated land (Table S1).

In addition, irrespective of the agroecological differences, the majority of the population of the study area relies on agriculture, with only a quarter engaged in non‐farm household income‐generating activities. On this background, population density relative to the total cultivable land size could also explain variation in the quantity of per capita agriculture production (Komarek & Msangi, 2019). Population density per hectare of available agricultural land is twice higher in the midland than that of the upper highland, at 1.63 and 0.84, respectively.

Carbohydrates contribute 77% or more to the total per capita energy production across all the AEZs. The share of dietary lipids is only 6–8%. Dehghan et al. (2017) indicated that energy intake from carbohydrates exceeding 60–70% is associated with a higher risk of major cardiovascular diseases and overall mortality. Therefore, along with increasing the availability of energy, agriculture production should allow a higher share of energy from non‐cereal crops and simultaneously supply optimal levels of essential nutrients.

The risk of vitamin A deficiency from supply was shown to decrease across the agroecological gradient, spanning from midland (46%), highland (39%), and the upper highland (31%). This could be because of increased per capita production of livestock products (see Figure 6). Livestock products, including whole milk and eggs, are good sources of highly bioavailable vitamin A (Alonso et al., 2019; Codjia, 2001). Per capita retinol equivalent in μg supplied from livestock products increased from 26.76 in the midland, 51.83 in the highland, to 64.34 in the upper highland (Table S2). The quantity of legumes produced is inversely related to per capita folate deficiency.

A per capita deficiency of folate is inversely associated with the quantity of legumes produced. Upper highlanders are 63% at risk of deficiency, whereas the major legume‐producing highlanders are presented with a <2% risk of folate deficiency. It has been indicated that low intake of dietary sources that are rich in folate, such as legumes and green leafy vegetables, is the primary cause of folate deficiency (Allen, 2008).

Supply deficits of Ca were found to be significantly high across all the AEZs. This could be multi‐factorial: (i) per capita availability of Ca‐rich foods like milk in a day is very low, ranging from 58 mL in midland to 140 mL in upper highland (Table S3); (ii) unlike the other minerals, the requirement of Ca is far higher to achieve. For instance, cereals provide a greater proportion of dietary Zn and Fe requirements than Ca. For this reason, Gashu et al. (2021) indicated that the dietary Ca requirement from the consumption of crops is likely to meet less than 25% of the required amount for most of the population; (iii) the local nutrient composition of major crops for Ca content in the present study is found to be lower than what has been used in previous studies that have estimated national and regional level supply side inadequacies (Baye et al., 2019; Sheehy et al., 2019). For instance, these studies used the concentrations of Ca for the two major crops, teff and wheat, of 126–180 and 34–46 mg/100 g, respectively. In the present study, the concentrations are far lower: teff (30.7 mg/100 g) and wheat (8.8 mg/100 g). Differences in crop nutrient concentrations have been well documented for variations across space and threshing methods (Gashu et al., 2021; Guja & Baye, 2018). In line with the present study, Weldehawaria (2021) reported that the Ca content of teff collected from multiple sites in the Amhara region was between 25.3 and 28.6 mg/100 g. Several other factors could hinder crop Ca availability from soil, including the concentration of Ca in soil and soil pH. Bevis and Hestrin (2021) showed that an increase in soil pH improves the relationship between the extractable soil Ca and crop Ca concentrations.

The prevalence of the per capita risk of Fe and Zn deficiency is generally lower across all the AEZs, which is below 10%. However, the available supply of minerals has been shown to be greatly affected by antinutrients. For most of the major crops, phytate‐to‐mineral molar ratios are not within the acceptable limit. Even though phytate concentrations are higher in teff among the studied crops, these phytate concentrations are lower than the 500 and 842 mg/100 g reported in teff by Legesse (2019) and Abebe et al. (2007), respectively.

Furthermore, phytate concentrations in teff have shown variations across the investigated AEZs. The mean phytate concentration of teff is 25% higher in midland (399 mg/100 g) than in highland (320 mg/100 g) at a *p*‐value = .016. Crop phytate concentrations have been demonstrated to vary greatly depending on variety and growing conditions, including soil phosphorus concentrations or its application rates (Bakhite et al., 2021; Schlemmer et al., 2009; Zaw Oo et al., 2023). According to the soil fertility status and fertilizer recommendation atlas of the Amhara region, the available phosphorus (mg/kg) in the soils of the study villages in the midland AEZ was classified as low, whereas it was very low in the highland villages (MoANR/ATA, 2016).

Similar variations in phytate concentrations have been observed in other crops as well. Rice phytate concentration varies by 8–125% depending on soil phosphorus application rate (Zaw Oo et al., 2023) and by 68% due to differences in variety (Legesse, 2019). Therefore, household food processing methods, including germination, soaking, or fermentation, are strongly needed to alleviate the adverse effects of antinutrients and enhance the bioavailability of essential dietary minerals from crops (Sandberg & Andlid, 2002). Fermenting flour to produce *injera* (a thin flat bread that is commonly consumed in Ethiopia) increases the bioavailability of mineral micronutrients by stimulating phytase enzymes to degrade and thereby reduce phytate concentrations in staple crops, including teff (Baye et al., 2014; Gabaza et al., 2018).

The present study observed that Ca concentrations in wheat and teff varied between the midland and highlands. Variations in mineral concentrations of the same crop [type] could arise due to spatial variation in soil and landscape factors and the effects of extrinsic soil dust (Gashu et al., 2021; Guja & Baye, 2018). Similar variation has also been observed in the protein concentration of wheat; about 2% higher wheat protein concentration has been observed in the midland compared with the highland. Johansson et al. (2020) indicated that variations in the protein concentration of wheat are largely attributed to changes in climatic factors, crop management, and genetic characteristics. In the present study, farmers across the two AEZs have used similar wheat varieties and comparable agriculture management practices, including the utilization of nitrogen‐based inorganic fertilizers (see Table 1).

In addition, among the climatic factors, the total monthly rainfall across the main wheat growing season (*meher)* was comparable (*p*‐value .699) between the two AEZs (Table S4). However, the mean monthly temperature has shown a significant difference (*p*‐value .016), with a mean difference of 4.3°C higher in midland AEZ across the main wheat growing season (Figure S2). In line with this, Zhou et al. (2021) indicated that temperature is the most dominant climatic factor responsible for the variability of protein concentration. They observed 0.75% to 1.08% higher protein concentrations for a unit increase in temperature (°C). Likewise, several studies have reported positive relationships between the higher temperature and protein concentration of wheat (Dupont et al., 2006; Liu et al., 2017; Nuttall et al., 2018). Therefore, utilization of single nutrient values from national or international databases may disregard variabilities arising in nutrient concentrations from various geographic contexts for estimating nutrient production.

The present study has strengths and limitations as well. Among its strengths, the study utilized food composition data for major crops from its own data collected locally for this purpose. The study was able to assess the agroecological‐based food production inadequacy of various nutrients, giving attention to predominantly rural areas that are highly vulnerable to undernourishment.

However, there are several restrictions that need to be considered when interpreting our results. First, we did not consider the role of the market, which is assumed to affect household food availability other than local‐level production (Sibhatu et al., 2015; Stifel & Minten, 2019). Second, our estimates consider agricultural food production, which does not always equate to consumption. Factors such as household food preparation methods and intra‐household distribution were not considered. Those can all affect what is actually consumed from what is produced (Coates et al., 2018; Gebremedhin et al., 2017; Hotz & Gibson, 2007). Third, the study considered crop yield data without considering post‐harvest losses. Consequently, in the future, a correction may be useful to apply.

Finally, the food supply chains between countries are challenged these days by several factors, including conflicts, the pandemic, and trade restrictions (Dyson et al., 2023; Galanakis, 2023; Gliessman, 2020). This necessitates a resilient national food supply that is adequate in terms of calories and essential nutrients at the individual level. This in turn requires auditing the local food production with up‐to‐date food composition data, preferably developed and applicable at the sub‐national level. Doing this could help to develop more reliable and context‐specific agriculture, nutrition, and health interventions. In this regard, the present study has contributed methods with field crop sampling for smallholding farms and has portrayed how to estimate individual‐level nutrient demands met in the context of local food production that considers several factors. We see it as an important opportunity to implement these findings in research, societal institutes, and governmental organizations for years to come.

## 5 CONCLUSIONS

Agroecology determines the choice of crop produced, and in this way, it affects the available supply of energy and nutrients. The nutrient composition of the same crops when grown in different agroecologies shows differences in composition. Agroecological variations should be a key consideration when designing food system interventions that aim to improve diets. No single agroecology can meet all requirements when relying solely on its production. This calls for production diversification and integrated food systems that allow market exchanges between agroecologies. Across all the AEZs, the household food processing method that employs fermentation would maximize the utilization of mineral micronutrients from major crops.

## AUTHOR CONTRIBUTIONS

**Habtamu Guja:** Conceptualization (equal); data curation (equal); formal analysis (equal); investigation (equal); methodology (equal); project administration (equal); supervision (equal); writing – original draft (equal); writing – review and editing (equal). **Mariana Belgiu:** Conceptualization (equal); data curation (equal); formal analysis (equal); methodology (equal); project administration (equal); supervision (equal); writing – review and editing (equal). **Lidya Embibel:** Conceptualization (supporting); data curation (equal); formal analysis (supporting); investigation (equal); methodology (equal); project administration (equal). **Kaleab Baye:** Conceptualization (equal); data curation (equal); formal analysis (equal); methodology (equal); project administration (equal); supervision (equal); writing – review and editing (equal). **Alfred Stein:** Conceptualization (equal); data curation (equal); formal analysis (equal); methodology (equal); project administration (equal); supervision (equal); writing – review and editing (equal).

## FUNDING INFORMATION

This study was supported by the Dutch Organization for Internationalization in Education (Nuffic), the University of Twente, Faculty of Geo‐information Science and Earth Observation, and the Ministry of Science and Higher Education of Ethiopia (MoSHE) under the Ethiopian Educational Network to Support Agriculture Transformation (EENSAT) project (CF13198, 2016).

## CONFLICT OF INTEREST STATEMENT

The authors declare no conflict of interest.

## ETHICAL APPROVAL

The study has received ethical approval from the institutional review board of the College of Natural and Computational Sciences, Addis Ababa University (Reference No: CNSDO/185/12/19).

## CONSENT TO PARTICIPATE

Informed consent was obtained from study participants prior to their inclusion in the study.

## Supporting information


Data S1.

Data S2.

Data S3.

## Data Availability

All of the data generated or analyzed during this study are included in this article and its supplementary information files. In case more data is needed for specific purposes, it is available from the corresponding author on request.
