# Supplementary material for: Examining energy and nutrient production across the different agroecological zones in rural Ethiopia using statistical methods
Source: Food Sci Nutr. 2023 Sep 15;11(12):7565–80. doi: 10.1002/fsn3.3676 (PMC10724589; doi:10.1002/fsn3.3676)
Supplement: Supplementary file 1 — Data S1. [file FSN3-11-7565-s001.docx]

**Examining energy and nutrient production across the different agroecological zones in rural Ethiopia using statistical methods**

*Food Science and Nutrition*

Habtamu Guja^1,2 *^, Mariana Belgiu^1^, Lidya Embibel^2^, Kaleab Baye^2^, Alfred Stein^1^

^1^Faculty of Geo-information Science and Earth Observation (ITC), University of Twente, Enschede, The Netherlands.

^2^Center for Food Science and Nutrition, College of Natural and Computational Sciences, Addis Ababa University, Addis Ababa, Ethiopia.

^*^Corresponding Author, email: habtamugujab@yahoo.com and habtamu.guja@aau.edu.et

**Supporting Information File 1 (SuppInfo_File_1)**


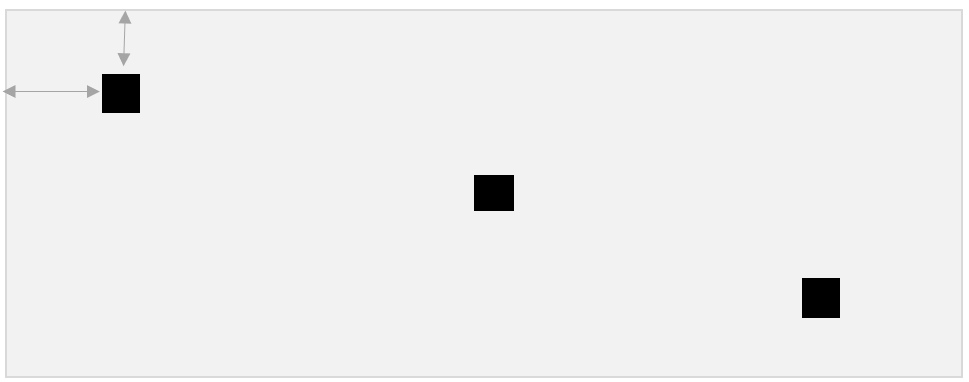


**FIGURE S1:** Diagonal sub-sampling protocol - target layout of the three subsamples (1m^2^ quadrant dark squares) for a standing crop in the field. Double arrow lines represent sampling distance away from the adjacent plot.

| **TABLE S1:** Productivity of major crops grown in South Wollo zone, 2020. | |
| --- | --- |
| Crop type | Productivity (kg/ha) |
| Sorghum | 2839 |
| Teff | 849 |
| Wheat | 1952 |
| Bean | 1125 |
| Barley | 1665 |

| **TABLE S2:** Share of cereals, legumes and livestock products to the total per capita energy & nutrient supplied across the agroecological zones (AEZs). | | | | | | | | |
| --- | --- | --- | --- | --- | --- | --- | --- | --- |
|  |  | **Midland** |  |  | **Highland** |  | **Upper highland** | |
|  | Cereals | Legumes | Livestock products | Cereals | Legumes | Livestock products | Cereals | Livestock products |
| Energy (kcal) | 1992.89 | 197.81 | 43.55 | 3156.89 | 212.18 | 83.92 | 4328.50 | 103.12 |
| Protein (g) | 47.19 | 13.55 | 1.98 | 80.19 | 14.54 | 3.83 | 103.06 | 4.76 |
| Fat (g) | 16.08 | 0.97 | 3.08 | 20.48 | 1.04 | 5.97 | 23.02 | 7.41 |
| Carbohydrate (g) | 399.82 | 29.99 | 1.97 | 631.89 | 32.17 | 3.71 | 869.39 | 4.35 |
| Fiber (g) | 30.45 | 7.00 | 0.00 | 61.30 | 7.51 | 0.00 | 112.01 | 0.00 |
| Ca (mg) | 117.55 | 17.13 | 34.32 | 111.85 | 18.37 | 66.43 | 168.24 | 82.47 |
| Fe (mg) | 32.86 | 2.41 | 0.58 | 53.57 | 2.59 | 1.13 | 104.10 | 1.40 |
| Zn (mg) | 13.59 | 1.57 | 0.22 | 23.94 | 1.69 | 0.43 | 38.14 | 0.53 |
| Vitamin A (µg RE) | 9.74 | 0.00 | 26.76 | 34.75 | 0.00 | 51.83 | 81.85 | 64.34 |
| Folic acid (µg) | 133.79 | 226.34 | 2.92 | 292.79 | 242.79 | 5.65 | 289.66 | 7.01 |
| Vitamin C (mg) | 3.15 | 0.77 | 1.20 | 6.25 | 0.83 | 2.30 | 6.08 | 2.80 |
| Livestock products: Egg, milk and honey. Cereal: Teff, wheat, sorghum & barley. Legume: common bean. | | | | | | | | |
| Carbohydrate (utilizable) (g)  µg RE: microgram retinol equivalent | | |  |  |  |  |  |  |

| **TABLE S3:** Per capita food production in gram by food group across the agroecological zones. | | | |  |  |  |  |  |  |
| --- | --- | --- | --- | --- | --- | --- | --- | --- | --- |
| **Food categories** | **ML** | **HL** | **UHL** |  |  |  |  |  |  |
| Grains, roots and tubers | 550.69 (78.18) | 888.08 (79.95) | 1235.25 (86.48) |  |  |  |  |  |  |
| Legumes and nuts | 60.83 (8.64) | 56.06 (4.92) | 0(0) |  |  |  |  |  |  |
| Dairy products | 57.73 (8.20) | 93.37 (8.32) | 140.76 (9.85) |  |  |  |  |  |  |
| Eggs | 8.86 (1.26) | 17.04 (1.50) | 5.78 (0.40) |  |  |  |  |  |  |
| Poultry, fish, meat **†** | 10.42 (1.48)) | 40.09 (3.40) | 19.3 (1.35) |  |  |  |  |  |  |
| Vitamin A rich fruits and vegetables **‡** | 4.33 (0.61) | 21.65 (1.90) | 4.33 (0.30) |  |  |  |  |  |  |
| Other fruits and vegetables **‡** | 11.50 (1.63) | 23.0 (2.02) | 23.0 (1.61) |  |  |  |  |  |  |
| **Total** | **704.36** | **1139.29** | **1428.42** |  |  |  |  |  |  |
| Values are per capital production per day in gram by food group, values in parentheses are percent  produced relative to other food groups in each AEZ. ML Midland, HL Highland, UHL Upper highland. | | | | | | | | | |
| **†** Flesh foods were estimated considering chicken, goat, and sheep population and using technical  conversion factors for agriculture commodities from FAO (1972). | | | | | | | | |  |
| **‡** Central Statistical agency (CSA, 2020). The Federal Democratic Republic of Ethiopia Central Statistical  Agency Agricultural Sample Survey 2020. Volume VII - Report on Crop and Livestock Product Utilization | | | | | | | | | |
| (Private Peasant Holdings, *Meher* Season). Statistical Bulletin 588, Addis Ababa, Ethiopia. | | | | | | | |  |  |
|  |  |  |  |  |  |  |  |  |  |

| **TABLE S4:** Climate and agriculture management across the main wheat growing season (*meher*) in midland and highland agroecological zones. | | | | |
| --- | --- | --- | --- | --- |
| **Variables** | **ML** | **HL** | **P-value** | **Mean Difference** |
| Temperature† | 19.56+2.43 | 15.25+2.19 | 0.016 | 4.30+1.33 |
| Precipitation† | 148.98+137.07 | 163.48+146.96 | 0.699 | -14.5+82.0 |
| Wheat variety | Danda’a (Danphie) | Danda’a (Danphie) |  |  |
| Recommended seed rate | 150 kg/ha | 150 kg/ha |  |  |
| Fertilizer type& app rate | NPSB+K | NPSB+K |  |  |
| † Non-parametric Mann-Whitney test was used to compare means*.* NPSB: 18.9N + 37.7 P_2_O_5_ + 6.95 S + 0.1 B.  K: KCl (Murite of Potash, 59% K), Urea (46% N). Mean difference = ML - HL  **Source:** Agriculture offices of the studied districts, MoANR/ATA (2016); NASA’s POWER project, 2020. | | | | |


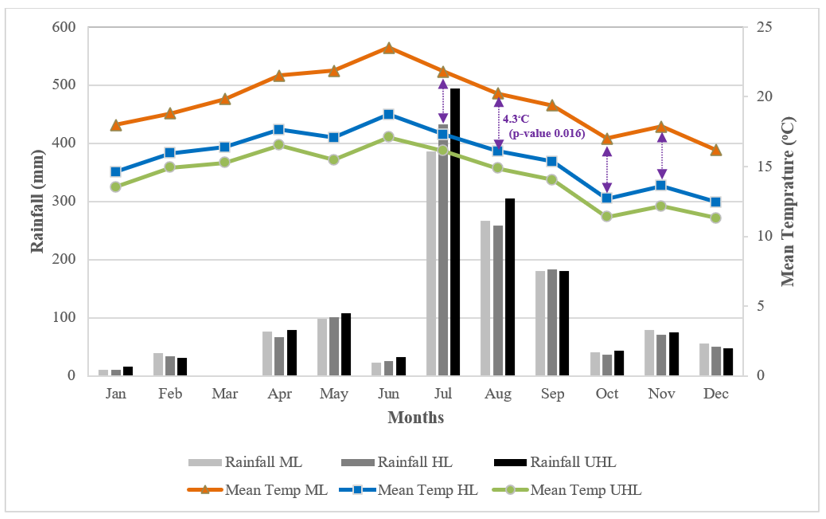


**FIGURE S2:** Monthly total rainfall and mean temperature of the study area by agroecological zones for the year 2021. ML Midland, HL Highland and UHL Upper highland. Monthly data obtained from a global perspective on renewable energy resources: NASA’s prediction of worldwide energy resources (POWER) project. <https://power.larc.nasa.gov/data-access-viewer/>
