# Supplementary material for: Examining energy and nutrient production across the different agroecological zones in rural Ethiopia using statistical methods
Source: Food Sci Nutr. 2023 Sep 15;11(12):7565–80. doi: 10.1002/fsn3.3676 (PMC10724589; doi:10.1002/fsn3.3676)
Supplement: Supplementary file 3 — Data S3. [file FSN3-11-7565-s003.docx]

**Examining energy and nutrient production across the different agroecological zones in rural Ethiopia using statistical methods**

*Food Science and Nutrition*

Habtamu Guja^1,2 *^, Mariana Belgiu^1^, Lidya Embibel^2^, Kaleab Baye^2^, Alfred Stein^1^

^1^Faculty of Geo-information Science and Earth Observation (ITC), University of Twente, Enschede, The Netherlands.

^2^Center for Food Science and Nutrition, College of Natural and Computational Sciences, Addis Ababa University, Addis Ababa, Ethiopia.

^*^Corresponding Author, email: habtamugujab@yahoo.com and habtamu.guja@aau.edu.et

**Supplementary material for review**

**(Graphical table of contents)**


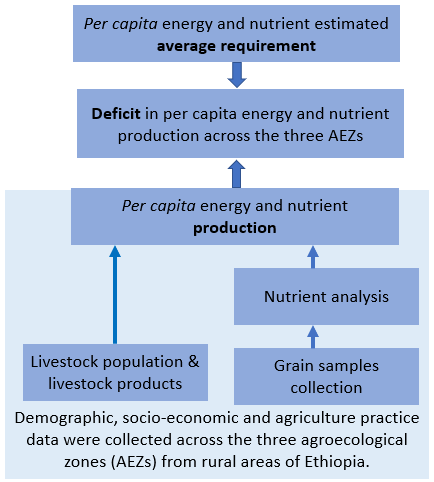


This research article reveals the inadequacies in nutrient availability across midland, highland and upper highland agroecological zones from several rural districts situated in Ethiopia.
